# Taste Receptors Mediate Sinonasal Immunity and Respiratory Disease

**DOI:** 10.3390/ijms18020437

**Published:** 2017-02-17

**Authors:** Jennifer E. Douglas, Noam A. Cohen

**Affiliations:** 1 Perelman School of Medicine, University of Pennsylvania, Philadelphia, PA 19104, USA; Jennifer.Douglas@uphs.upenn.edu; 2 Monell Chemical Senses Center, Philadelphia, PA 19104, USA; 3 Department of Otorhinolaryngology–Head and Neck Surgery, University of Pennsylvania Health System, Philadelphia, PA 19104, USA; 4 Philadelphia Veterans Affairs Medical Center Surgical Services, Philadelphia, PA 19104, USA

**Keywords:** taste receptors, chronic rhinosinusitis, mucociliary clearance, airway physiology, biofilm, innate immunity, upper respiratory infection

## Abstract

The bitter taste receptor T2R38 has been shown to play a role in the pathogenesis of chronic rhinosinusitis (CRS), where the receptor functions to enhance upper respiratory innate immunity through a triad of beneficial immune responses. Individuals with a functional version of T2R38 are tasters for the bitter compound phenylthiocarbamide (PTC) and exhibit an anti-microbial response in the upper airway to certain invading pathogens, while those individuals with a non-functional version of the receptor are PTC non-tasters and lack this beneficial response. The clinical ramifications are significant, with the non-taster genotype being an independent risk factor for CRS requiring surgery, poor quality-of-life (QOL) improvements post-operatively, and decreased rhinologic QOL in patients with cystic fibrosis. Furthermore, indirect evidence suggests that non-tasters also have a larger burden of biofilm formation. This new data may influence the clinical management of patients with infectious conditions affecting the upper respiratory tract and possibly at other mucosal sites throughout the body.

## 1. Introduction

The upper airway is constantly exposed to a number of pathogens, toxins, and other irritative particulates that are typically successfully defended against by the upper airway innate immune defenses. Recently, the bitter taste system, far from its site of original identification in taste buds, has been implicated in this defense pathway with implications for the pathogenesis of upper respiratory infectious/inflammatory diseases and biofilm formation. This review will present recent evidence for the role of the bitter taste receptor T2R38 in chronic rhinosinusitis (CRS) and put forth support for an expanded role for individual taste differences in the clinical management of patients with upper respiratory infections.

**Figure 1:**
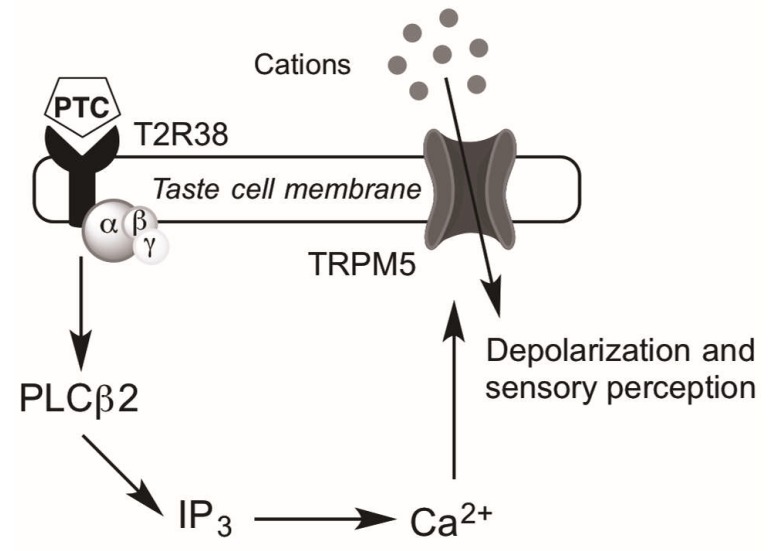
Intracellular taste receptor signaling. Binding of the bitter compound phenylthiocarbamide (PTC) to the T2R38 bitter taste receptor in sinonasal epithelial cells results in activation of an undetermined G-protein that then activates phosopholipase C isoform β2 (PLCβ2), resulting in increased inositol 1,4,5-trisphosphate (IP_3_) [16]. IP_3_ induces the release of calcium (Ca^2+^) from the endoplasmic reticulum. Ca^2+^-dependent activation of TRPM5 channels (transient receptor potential cation channel subfamily M member 5) depolarizes the membrane and results in bitterness perception [1,17,18].

Bitter taste is one of five unique tastes in addition to salty, sour, sweet, and umami that humans are capable of perceiving. Receptors for each of these tastes are present in the oral cavity, where bitter taste receptors (T2Rs) specifically signal the ingestion of potentially toxic substances and mediate aversive behavior [1]. As G protein-coupled receptors (GPCRs), T2Rs feature seven transmembrane domains but are unique in having a short extracellular N-terminus, in contrast with other taste receptors (e.g., T1R sweet taste receptors) [2,3,4]. Recently, T2Rs have also been identified in extraoral sites including, but not limited to, the upper and lower respiratory tracts, skin, thyroid, gastrointestinal tract, and testes [1,5,6,7,8,9,10]. Within the airway, the bitter taste receptor T2R38 has specifically been identified on ciliated epithelial cells [11,12,13]. T2Rs in upper respiratory cells appear to utilize most of the canonical bitter taste signaling cascade including phospholipase C β2 and TRPM5 (transient receptor potential cation channel subfamily M member 5), but interestingly not gustducin, the G-protein classically associated with T2Rs in the tongue (Figure 1) [11,14]. In the airway, a ligand for the human T2R38 appears to be acyl-homoserine lactones (AHLs), quorum sensing molecules secreted by gram-negative organisms [1]. Additionally, the extraoral expression of T2Rs has been hypothesized to cause many of the poorly understood off target effects of many medications, which are often bitter in taste [15].

The bitter taste receptor family includes approximately 25 different T2Rs, each of which is encoded by a corresponding bitter taste receptor gene (*TAS2R*s) [19]. One of the most well-studied receptors among this group is the bitter taste receptor T2R38, which is encoded by the *TAS2R38* gene and was first characterized molecularly in 2005 [20]. It is specifically responsive to the bitter compounds phenylthiocarbamide (PTC), propylthiouracil (PROP), the plant compound goitrin (common in cruciferous vegetables), and other chemically similar substances [21]. Prior studies show that *TAS2R38* exists in two common haplotypes that are either functional and respond to its bitter agonists, or are non-functional and are not activated by its bitter agonists, based on three missense single nucleotide polymorphisms (SNPs) [20]. The specific coding logic is further detailed below. Many common bitter foods such as broccoli, Brussels sprouts, coffee, and beer contain compounds that agonize T2R38 and as such, genetic variability in *TAS2R38* influences dietary preferences through differences in psychophysical bitterness perception [22]. Further, the extraoral expression of T2R38 has been shown to influence upper respiratory immunity with clinically significant effects on CRS [14,23]. In the paragraphs below, we discuss the state of knowledge on the expression pattern of T2R38 in the upper respiratory epithelium, its role in the pathogenesis of CRS and other respiratory conditions, the emerging understanding of its influence on biofilm formation, and the implications for clinical treatment.

## 2. Genetic Variability of *TAS2R38*

As previously mentioned, the *TAS2R38* gene features two common haplotypes that confer significant phenotypic variability in bitterness sensitivity. There exist three SNPs within the gene that each produce an amino acid change (P49A, A262V, and V296I), resulting in two common haplotypes: a proline-alanine-valine (PAV) haplotype that is exquisitely sensitive to PTC due to successful signal transduction and intracellular calcium release (Figure 1), and an alanine-valine-isoleucine (AVI) haplotype that is relatively insensitive to PTC due to an absence of signal transduction. Thus, individuals can either be homozygous for the PAV allele (so-called “tasters” for their ability to taste PTC), homozygous for the AVI allele (“non-tasters” for their relative inability to taste PTC), or heterozygous (intermediate tasters with variable PTC sensitivity) [8]. Importantly, the AVI haplotype exists in a significant portion of the population, with frequency ranging from zero to 66.7% in various subgroups [24]. Of note, there are three less common *TAS2R38* haplotypes (AAI (alanine-alanine-isoleucine), PVI (proline-valine-isoleucine), and AAV (alanine-alanine-valine)), each of which show intermediate sensitivity to PTC; however, these sub-types make-up only 1%–5% of the Caucasian population and up to 20% of the African American population and will not be further discussed here [20,25].

## 3. Mechanisms of Upper Airway Immunity

**Figure 2:**
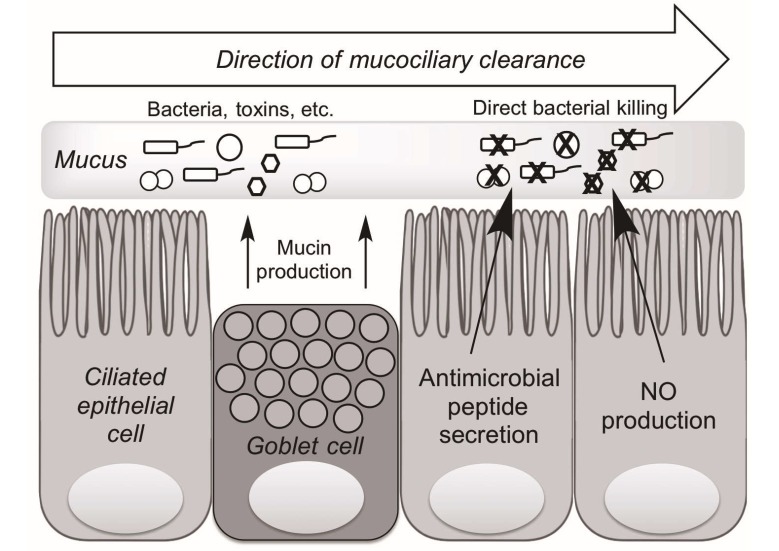
Mechanisms of upper airway innate immunity. Ciliated epithelial and goblet cells work in concert to rid the airway epithelium of foreign pathogens and other toxins through a process known as mucociliary clearance (MCC). Goblet cells secrete mucin that physically traps bacteria and other toxins while ciliated epithelial cells beat in a coordinated fashion to expel trapped pathogens from the airway. Ciliated cells also produce antimicrobial peptides and nitric oxide (NO), which both are directly bactericidal. NO also results in increased ciliary beat frequency, enhancing MCC.

There are two primary cell types within the upper airway epithelium, goblet cells and ciliated cells, which work synergistically to keep the mucosa clean. Goblet cells, which produce mucin, a proteinaceous substance that physically traps pathogens and other foreign particles within the airway surface liquid (ASL), and ciliated cells, which beat in a coordinated fashion to propel mucin out of the airway [26,27,28,29]. Together, these cells contribute to the crucial process of mucociliary clearance (MCC) that physically clears the area of trapped pathogens and particles (Figure 2).

Additionally, the epithelium produces a number of compounds that enhance the local immune response. Specifically, ciliated cells produce antimicrobial peptides (AMPs) as well as nitric oxide (NO) that work to inhibit pathogen colonization [30]. These peptides include defensins, lactoferrin, and cathelicidins. β-defensin 1 and 2, specifically, are effective against both gram-positive and gram-negative bacteria, with particular potency against gram-negative bacteria such as *Pseudomonas aeruginosa* and *Klebsiella pneumonia* [14]. Nitric oxide has parallel benefits: local increase in NO concentration enhances the process of MCC by increasing ciliary beat frequency (CBF), while also inducing direct DNA damage as a reactive oxygen species, leading to bacterial cell death [11].

Bacteria and other pathogens like fungi have naturally developed mechanisms to evade these immune responses and secrete compounds including pyocyanin and pyoverdin that paralyze cilia, or aflatoxin that slows cilia, thereby dismantling the crucial process of MCC [31,32]. Additionally, gram-negative microbes also secrete a class of compounds known as AHLs that communicate within a bacterial community to report microbial density, thereby coordinating virulence through biofilm formation, toxin secretion, and acquisition of antibiotic resistance [33,34]. Biofilms are a particularly challenging form of infection to treat as they represent a coalescence of single-cell, planktonic bacteria into a bacterial community with a glycocalyx scaffold that increases bacterial adherence, limits antibiotic penetration, and prevents phagocytosis by immune cells. Additionally, biofilms provide a chronic source of shedding bacteria, toxins, and antigens that stimulate the immune system and generate persistent localized inflammation. In patients with CRS, biofilms have been associated with persistent infections and poor treatment outcomes [35,36,37].

## 4. Taste Receptors and Upper Airway Immunity

As with the signaling pathway in taste buds, stimulation of taste receptors on airway cells with bitter agonists like denatonium benzoate (DB) induces an intracellular calcium release [14]. Additionally, early studies found that murine solitary chemosensory cells (SCCs) respond directly to AHLs, but the exact T2R responsible for this activation was undetermined [38]. Thus, early work demonstrated that non-ciliated murine nasal cells that express bitter taste signaling proteins are activated by gram-negative quorum-sensing molecules. 

Our laboratory has utilized a culture technique known as the air-liquid interface culture (ALI) to facilitate a better understanding of these pathways in human upper airway immunity [39,40]. The technique recapitulates the natural polarization of the airway epithelium, enabling in vitro modeling of cell activation, signaling, and response to pathogen invasion [11,14].

Investigating human sinonasal epithelial cultures, our group demonstrated that the gram-negative AHLs *N*-butyryl-l-homoserine lactone (C4HSL) and *N*-3-oxo-dodecanoyl-l-homoserine lactone (C12HSL) activate T2R38, which in the human is exclusively expressed in ciliated cells, not SCCs [11,41]. Stimulation of T2R38 in human ciliated cell leads to intracellular calcium release and activation of nitric oxide synthase leading to the production of NO [11]. The NO diffuses across the cell membrane into the overlying mucus and has direct bactericidal activity. [42,43,44,45]. Additionally, NO triggers an increase in CBF, promoting removal of offending pathogens from the airway [46,47]. Importantly, NO production by activation of the functional (PAV) T2R38 occurs at physiologic concentrations of AHLs (1–10 µM), as evidenced by in vitro experiments comparing the response of ALIs to conditioned media from *P. aeruginosa* either with or without the capability of producing AHLs [11]. Further, this process occurs through a pathway consistent with what is known of intracellular taste receptor signal transduction, producing activation of phosopholipase C isoform β2 (PLCβ2) and the non-selective cation channel, TRPM5 [15,16,48]. This pathway occurs in a genotype-dependent manner akin to that in taste buds; T2R38-AVI individuals do not exhibit the crucial NO response to the gram-negative AHLs [14]. Thus, it follows that non-tasters might be at increased risk of gram-negative bacterial invasion and persistence, which may contribute to T2R specific alterations in the upper respiratory microbiome.

**Figure 3:**
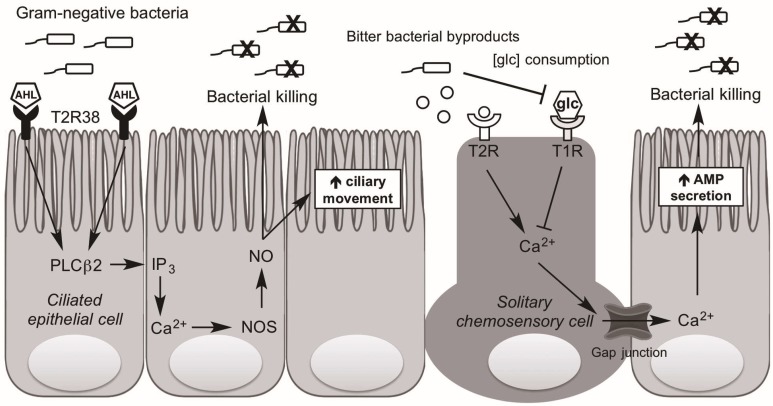
Taste receptor-dependent upper airway immunity. Ciliated epithelial cells express T2R38 while solitary chemosensory cells (SCCs) express both T2Rs and T1Rs. Gram-negative bacteria produce acyl-homoserine lactones (AHLs), which bind to and activate T2R38, producing an intracellular cascade activating PLCβ2 and production of IP_3_. This increases nitric oxide (NO) production through the activation of nitric oxide synthase (NOS), which both directly kills bacteria and enhances ciliary beating. Additionally, presumed other bitter bacterial byproducts activate an as yet unknown T2R(s) on solitary chemosensory cells and, through typical taste receptor intracellular signaling including gustducin, yields increased Ca^2+^. This Ca^2+^ diffuses into adjacent ciliated cells via gap junctions where it produces increased antimicrobial peptide (AMP) secretion, killing pathogens. Due to glucose (glc) consumption by bacteria, microbial colonization also decreases the T1R-mediated inhibition of SCC Ca^2+^ release, thereby contributing to enhanced antimicrobial peptide production.

While AHLs in the human nose stimulate T2Rs on ciliated cells to activate NO production, AHLs in the mouse nose stimulate T2Rs on SCCs (discrete non-ciliated cells) to induce a cholinergic-mediated neurogenic inflammatory response [38,49,50]. While acetylcholine release has not been demonstrated following stimulation of human SCCs, in vitro studies have found that activation of T2Rs present on human SCCs by DB and other bitter tasting compounds such as absinthin, parthenolide, and amoraogentin results in a release of intracellular Ca^2+^, which propagates to the surrounding epithelial cells via gap junctions and stimulates release of AMP stores [14] (Figure 3). Significantly, this immune activation does not occur with AHL stimulation of human SCCs. It is hypothesized that an as yet unidentified bacterial product/byproduct triggers T2Rs on human SCCs to activate this robust antimicrobial defense pathway.

Interestingly, T1R2+3 sweet taste receptors are also present on SCCs, where they are influenced in parallel by the presence of bacteria. Glucose is present in the airway due to a physiological “leak” across the epithelium [51]. Upon glucose binding the SCC sweet taste receptor, Ca^2+^ release is blocked, leading to decreased AMP release [14]. During microbial invasion, glucose concentration is decreased due to bacterial consumption. Thus, in the presence of local bacterial overgrowth/infection, the tonic sweet taste receptor brake on SCC activity is relieved, yielding local antimicrobial peptide secretion and reduction in the local microbes (Figure 3). Additionally, studies using sweet receptor antagonists such as lactisole demonstrate the specific inhibition in AMP release by the sweet receptor [14,52,53], while glucose transport inhibitors such as phloretin and phlorizin do not [14]. Clinically, this pathway has important implications, as individuals with diseases of glucose homeostasis such as diabetes mellitus have chronically elevated ASL glucose, and are known to suffer from a greater frequency of respiratory infections than patients without diabetes [54,55]. Diabetic patients with CRS also exhibit smaller improvements in QOL measures following sinus surgery [56].

## 5. Conditions of Defective Airway Immunity

**Figure 4:**
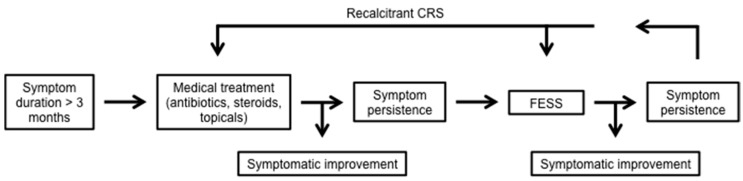
Treatment algorithm for chronic rhinosinusitis (CRS). CRS is diagnosed after symptoms, including headache, hyposmia, rhinorrhea, and nasal congestion persist for greater than three months. Treatment is initiated medically with oral antibiotics and steroids as well as topical steroids and nasal irrigation. Should symptoms persist, patients are offered surgical intervention with functional endoscopic sinus surgery (FESS). Continued symptoms of recalcitrant CRS are managed with repeated courses of culture-directed antibiotics, steroids, and topical treatment with revision sinus surgery when indicated.

Numerous diseases exist for which the pathways of airway immunity are important. We will discuss two of these conditions: CRS, which is a common syndrome effecting the upper airway and paranasal sinuses, and cystic fibrosis (CF), a disease affecting both the upper and lower airways. Patients suffering from CRS experience significant inflammation of the upper respiratory epithelium, with a resultant overproduction of mucin and a defect in MCC. Symptoms can include rhinorrhea, hyposmia, headaches, nasal obstruction, and facial pressure/pain. When these symptoms persist for three months or more and on a physical exam there are objective findings of nasal purulence or polyps, or paranasal sinus opacification on radiographic imaging, patients are given the diagnosis of CRS. The syndrome of CRS results in significant decrements in patient quality of life (QOL), as measured by the Sinonasal Outcome (SNOT-22) test, with individuals reporting lower QOL measures than patients with serious heart and lung diseases [57,58]. Treatment for CRS is initially medical, with courses of culture directed antibiotics, potent anti-inflammatories such as steroids, as well as topical irrigation with saline and/or steroid solutions. When symptoms persist despite medical therapy, patients are offered a surgical intervention known as functional endoscopic sinus surgery (FESS) to ventilate and drain the sinuses as well as optimize the exposure of the sinonasal epithelium to topical treatments (Figure 4).

While gram-positive bacteria such as *Streptococcus pneumoniae* are most frequently responsible for sinusitis and particularly acute sinusitis, recalcitrant CRS is more commonly linked with sinonasal biofilm formation and gram-negative bacteria such as *P. aeruginosa*, making antibiotic choice for coverage and penetration more complex [31]. The pathway mediated by bitter taste receptors in upper airway immunity directly influences these features characteristic of CRS.

Clinical studies first employed a retrospective analysis evaluating the frequency of *TAS2R38* genotypes within the non-polypoid CRS population undergoing FESS. Results found a disproportionate number of non-tasters (*TAS2R38*-AVI/AVI genotype) within the population, demonstrating that tasters (*TAS2R38*-PAV/PAV) are less likely to require surgical intervention for their CRS, likely due to enhanced upper airway immunity [59]. A follow-up prospective study confirmed that the non-taster genotype is an independent risk factor for CRS requiring surgical intervention [23]. More recently, a prospective study assessing post-operative improvement in SNOT-22 scores found that homozygous tasters experienced greater improvement in scores at one, three, and six months post-operatively as compared with heterozygotes and homozygous non-tasters [60]. Similar observations have recently been confirmed in a Canadian [61] and Polish cohort of patients [62]. However, an Italian cohort found no association between *TAS2R38* genotype and CRS either with or without polyps [63]. A significant limitation of this study is its small non-polyp population (*n* = 17), given that the genotype relationship has been previously shown to be exclusive to this CRS sub-group.

CF, in contrast, is an autosomal recessive genetic disorder caused by defective transcellular ion transport across the Cystic Fibrosis Transmembrane Conductance Regulator (CFTR), which is encoded by the *CFTR* gene. The disease is most commonly caused by the ΔF508 mutation but can be caused by one of almost 200 different mutations. [64,65]. While the preponderance of symptoms manifest in the lower airway epithelium, the disease affects all mucosal surfaces and nearly 50% of CF patients also suffer from CRS, with one-third of who require surgical management [66,67,68,69]. Because CF is characterized by a preponderance of *P. aeruginosa* colonization and biofilm formation, a link with bitter taste receptors has been hypothesized. A retrospective analysis identified that *TAS2R38* genotype correlates with both SNOT-22 scores and rhinologic-specific symptoms in CF patients [70]. Further, as the understanding of taste receptors in biofilm formation and gram-negative respiratory infections grows, an even more significant role for *TAS2R38* genotype in CF may be revealed.

## 6. Taste Influence on Biofilm

Biofilms not only influence CRS and CF, but also contribute significantly to treatment-resistant infections throughout the body [37,71]. Recently, a study sought to identify whether PTC sensitivity, as a proxy for *TAS2R38* genotype, is linked with biofilm formation based on the understanding of the role of T2R38 in upper airway immunity. Endoscopic nasal swabs were obtained from patients with CRS both with and without polyps, and analyzed using the Calgary biofilm detection assay. Results found an inverse linear relationship between biofilm formation and PTC sensitivity, indicating that patients with poor bitterness sensitivity (decreased T2R38 function) yielded more ex-vivo biofilm biomass [72]. While this correlation was true for the entire CRS cohort of patients, it was exclusively driven by those patients without polyps, likely due to different immunologic pathways driving polyp and non-polyp CRS [73]. Additionally, by using PTC taste sensitivity rather than *TAS2R38* genotype, this study is a proof of concept for the idea that a simple taste test can easily approximate genotype, simplifying the costly and time-consuming process of genotyping. However, further studies are necessary to definitively prove that bitterness sensitivity and expression of T2R38 in taste buds directly correlate with that in the nasal epithelium.

## 7. Conclusions

The understanding of the role of the bitter taste receptors in upper respiratory immunity continues to grow. What is clear is that bitter taste receptors, specifically T2R38, are expressed in the upper airway epithelium where they respond to bitter compounds produced by invading bacteria to potentiate the local innate immune response. Due to common individual genetic variation, non-taster individuals do not benefit from this taste receptor-dependent pathway of upper airway immunity and are at increased risk of gram-negative upper respiratory infections and non-polypoid CRS. Because of the role that biofilms play in the pathogenesis of recalcitrant respiratory disease, which has been directly correlated with *TAS2R38* genotype, it is likely that broader conclusions may be drawn about local immune responses at diverse sites throughout the body.

Going forward, taste receptors may be targeted for topical therapeutic intervention by using bitter compounds to directly activate the sinonasal bitter taste receptors. One could argue that a bitter taste panel may guide the ideal bitter compound(s) for individualized therapeutic intervention. This precision medicine could optimize individual treatment responsiveness, decreasing the use of oral steroids and antibiotics, limiting their contribution to the growing epidemic of antibiotic resistance [74]. Interestingly, this pathway has been largely tied to gram-negative bacteria. However, gram-positive bacteria are responsible for the large majority of CRS cases and the common pathogen *S. aureus* has been found to induce NO production in human upper airway epithelium, though through a process that is independent of T2R38 [75]. A better understanding of this process would have implications for the treatment of both acute and chronic sinusitis.

Additionally, future studies should aim to directly correlate the expression of T2R38 in the oral and nasal epithelium, as well as to assess the feasibility of instilling bitter compounds in nasal lavages or sprays to evaluate the benefit of taste receptor-targeted topical therapies. Because the T2R38 bitter taste receptor is one of only 25 different bitter taste receptors, it is necessary to understand how all bitter taste receptors, the so-called “bitterome”, work in concert to influence upper airway innate defenses [76]. By contrast with the bitter taste system, the sweet taste system remains relatively poorly understood and presents an opportunity for significant advances in understanding. With this knowledge, it is likely that the principles first identified and characterized within the sinonasal epithelium will become applicable to many mucosal sites throughout the body, with implications for the use of extraoral taste receptors in the treatment of a diverse group of infectious processes.

## Abbreviations

CRS	Chronic rhinosinusitis
GPCRs	G protein-coupled receptors
AHLs	Acyl-homoserine lactones
PTC	Phenylthiocarbamide
QOL	Quality of life
PLCβ2	Phosopholipase C isoform β2
IP_3_	Inositol 1,4,5-trisphosphate
Ca^2+^	Calcium ions
TRPM5	Transient receptor potential cation channel subfamily M member 5
PROP	Propylthiouracil
SNPs	Single nucleotide polymorphisms
PAV	Proline-alanine-valine *TAS2R38* haplotype (“tasters”)
AVI	Alanine-valine-isoleucine *TAS2R38* haplotype (“non-tasters”)
ASL	Airway surface liquid
MCC	Mucociliary clearance
NO	Nitric oxide
AMPs	Antimicrobial peptides
CBF	Ciliary beat frequency
DB	Denatonium benzoate
SCCs	Solitary chemosensory cells
ALI	Air-liquid interface culture
C4HSL	*N*-butyryl-l-homoserine lactone
C12HSL	*N*-3-oxo-dodecanoyl-l-homoserine lactone
glc	Glucose
CF	Cystic fibrosis
SNOT-22	Sinonasal outcome test
FESS	Functional endoscopic sinus surgery
